# The accuracy of fine-needle aspiration cytology and ultrasonography in assessing thyroid nodules in correlation with histopathology: a retrospective study

**DOI:** 10.1097/MS9.0000000000002676

**Published:** 2024-10-23

**Authors:** Mana Alhajlan, Mohammed Al-Masabi, Mohammed Al Mansour, Abdullah Saihb, Salem AlAyed, Rakan Alwadai, Abdullah Alhamami, Abdullah Alzarra, Mohammed Almarzooq, Faisal Ahmed

**Affiliations:** a Department of General Surgery, King Khalid Hospital, Najran, Saudi Arabia; b Operating Rooms Specialist, King Khalid Hospital, Najran, Saudi Arabia; c Department of Otorhinolaryngology, King Khalid Hospital, Najran, Saudi Arabia; d Department of Urology, School of Medicine, Ibb University, Ibb, Yemen

**Keywords:** accuracy, fine-needle aspiration cytology, goiter, thyroid malignancy, thyroid nodule, ultrasonography

## Abstract

**Background:**

Accurately diagnosing thyroid nodules is vital for preventing unnecessary surgeries and providing prompt therapy. Although fine-needle aspiration cytology (FNAC) and ultrasonography (US) are widely used diagnostic methods, their reliability is questioned. This study investigates the effectiveness of US and FNAC in thyroid nodule diagnosis and differentiates benign from malignant nodules in relation to final histopathological diagnosis.

**Method:**

A retrospective study including 307 adult patients with thyroid diseases who underwent neck US and FNAC before surgery was conducted between April 2019 and May 2023. The diagnostic efficacy of US, FNAC, and their combination usage was compared to histopathological results.

**Result:**

Histopathological findings revealed that 187 (61%) cases were benign, while 120 (39%) were malignant. The US features of ‘taller-than-wider’ forms and hypoechoic appearance had the highest diagnostic accuracy in characterizing malignant thyroid nodules, with 83 and 73% accuracy, respectively. The combination of US parameters demonstrated high sensitivity, specificity, positive predictive value, and negative predictive value (NPV) of 88.33, 63.10, 60.6, and 89.4%, with a statistically significant area under the ROC curve (AUC: 0.828, *P*<0.001) than individual parameters. FNAC’s sensitivity, specificity, PPV NPV, and accuracy in detecting malignant lesions were 50, 95, 86, 75, and 77%, respectively, with acceptable discrimination and statistical significance (AUC: 0.723, *P*<0.0001). The combination of US parameters and FNAC significantly improved the AUC value (AUC: 0.878, *P*<0.0001), sensitivity (83.33%), and specificity (79.14%). Univariate analysis showed that hypoechoic appearance, heterogenicity, large mass size (>4 cm), ‘taller-than-wider’, infiltrative margins, and microcalcifications were risk factors for malignancy in thyroid nodules and were statistically significant (all *P*-values <0.05).

**Conclusion:**

Combining US characteristics with FNAC results can afford the maximum analytical accuracy in distinguishing benign from malignant thyroid nodules. This strategy is practical due to its simplicity, minimal invasiveness, and cost-effectiveness, enabling robust management regimens and avoiding additional surgical procedures.

## Introduction

Highlights

- Thyroid nodules are a prevalent concern in clinical practice.
- FNAB is the most accurate procedure for differentiating benign from malignant thyroid nodules.
- Combined USG and FNAC can accurately differentiate benign and malignant thyroid nodules and avoid additional surgical procedures.
- Nondiagnostic rates may fall as expertise with USG-guided thyroid biopsies increases.

Thyroid nodules are common in surgical practice, affecting 4–5% of adults and up to 40% of those over 60^1^. The incidental detection of thyroid nodules ranges from 10 to 41%. The probability of malignant transformation in thyroid nodules ranges from 7 to 15%, and additional investigations are needed for exclusion and more effective treatment, with papillary carcinoma being the most prevalent histologic type^2^.

Ultrasonography (US) is a popular technique for thyroid nodule evolution. US features favoring malignancy are hypoechogenicity, irregularity, microcalcifications, solid nodules, a length longer than the width, and increased nodule vascularity on Doppler US^3^. The thyroid imaging reporting and data system (TI-RADS) is used to classify thyroid nodules as benign and malignant, with a sensitivity and specificity of 79 and 71%^4,5^. However, US accuracy is questioned due to the equipment’s sensitivity and the operator’s familiarity with the process^6^.

Thyroid nodules with abnormal findings in the US are highly recommended for fine-needle aspiration cytology (FNAC), a primary diagnostic test for thyroid nodules^3^. The most recent thyroid cytopathology guidelines employ the Bethesda System for Reporting Thyroid Cytopathology (TBSRTC) for the categorization scheme^3,7^. It classified the reporting system into six distinct diagnostic criteria, each with a different risk of malignancy^8^. The effectiveness of FNAC is dependent on competent aspiration, cytological interpretation, and rational analysis of cytological and clinical data. FNAC may mask radiological assessment, cause acellular smears, and have varying inadequacy rates due to operator dependence^3^. Few reports evaluate thyroid nodules with integrated US, FNAC, and their combination in correlation with histopathology^9–11^. This study investigates the effectiveness of US and FNAC in thyroid nodule diagnosis and differentiates benign from malignant nodules in relation to final histopathological diagnosis.

## Material and methods

A retrospective study involving 307 adult patients with thyroid diseases who underwent thyroid surgery was conducted between April 2019 and May 2023. The ethics committees approved the study. We conducted the survey following the Helsinki Declaration, and all patients provided informed consent before data capturing. The requirement for participant consent was waived due to the study’s retrospective nature and the absence of identifiable information. The manuscript has been reported per the strengthening the reporting of cohort, cross-sectional, and case–control studies in surgery (STROCSS) criteria^12^.

## Inclusion criteria

All patients with thyroid diseases who underwent neck US, followed by FNAC, and then thyroid gland surgery were included. This includes patients with suspected thyroid nodules for malignancy in the US, such as a diameter larger than 1.0 cm, extracapsular growth, enlarged cervical lymph nodes, a history of neck irradiation, or a history of thyroid malignancy in first-degree relatives’ degree, previous thyroid surgery for cancer, and increased calcitonin levels, or those candidates for surgery due to goiter and provided consent.

## Exclusion criteria

Patients with uncooperative or psychological diseases, patients with severe bleeding disorders [International ratio (INR)>1.4, prothrombin time (PT)>15 s, platelet count>50 000/mm^3^), and hot nodules on scintigraphy were excluded. We also excluded participants without US or FNAC assessments, those with missing histology data, and those who had previously undergone head and neck oncology surgery.

### Data collection

The collected data include age, sex, comorbidities, history of thyroid surgery, US report, FNAC cytology report, type of surgery, postoperative complications, and need for postoperative supplement therapy.

### US findings

The 2015 ATA Guidelines introduced a US scoring system that linked thyroid nodules to cancer risk based on their sonographic pattern and was used for US evaluation^13^. The US evaluation of thyroid nodules in benign versus malignancy features that included an assessment of their size (less than 4 cm vs. more than 4 cm), nodule echogenicity (hyperechoic vs. hypoechoic), composition (cystic or spongiform vs. solid), homogeneity (homogenous vs. heterogenous), width (wider-than-tall vs. taller than wider), margin status (regular border vs. irregular nodule borders), microcalcifications (negative or large comet tail artifacts vs. peripheral/rim calcifications), and thyroid extension (no thyroidal extension vs. extrathyroidal extension)^3^. We could not report these features by the TI-RADS, as we did not have all criteria in all cases.

### FNAC findings

The FNAC was performed under US supervision or by palpation without imaging, and the cytological diagnosis was classified using the Bethesda System.

Bethesda’s cytopathology reports were categorized into six types: Bethesda I, II, III, IV, V, and VI represent unsatisfactory material, benign, atypical/follicular lesion, suspected follicular neoplasia, suspected malignancy, and malignancy, respectively^3,7^.

### Postoperative histopathological findings

The postoperative histopathological result was reported and divided into benign lesions (including goiter, toxic multinodular goiter, thyroiditis (graves, Hashimoto’s), thyroid cyst, benign thyroid nodule, and follicular nodule) and malignant lesions (including follicular thyroid neoplasm, medullary thyroid neoplasm, papillary thyroid cancer, Hurthle cell neoplasm, lymphoma, carcinoma with mixed features, and metastatic carcinoma). The postoperative histopathological findings were classified as benign and malignant lesions and used as a golden standard for comparison.

### Primary outcome

The main outcome was to examine the diagnostic utility of FNAC and US findings individually and in combination compared to the postoperative histopathological diagnosis of thyroid nodules in terms of sensitivity, specificity, positive predictive value (PPV), negative predictive value (NPV), accuracy, and area under curve (AUC).

#### Statistical analysis

Continuous data was presented as mean and SD, while categorical data was presented as numbers (%). We assessed the sensitivity, specificity, positive predictive value (PPV), negative predictive value (NPV), accuracy, and rate of discordance of US and FNAC in detecting malignant and benign lesions, with sensitivity based on malignant detection and specificity based on benign lesions. Categorical variables were analyzed using the *χ* ^2^ test or Fisher’s exact test, whereas continuous variables were evaluated using Student’s *t*-test or the Mann–Whitney *U* test. Furthermore, a receiver operating characteristic (ROC) curve was developed to assess the diagnostic efficacy of US characteristics alone and in combination with FNAC in predicting malignancy. An area under cure (AUC) of 0.5 indicates no discrimination (i.e. the capacity to diagnose patients with and without the disease or condition based on the test); 0.7 to 0.8 was acceptable, 0.8 to 0.9 was excellent, and more than 0.9 was exceptional. Statistical significance was set at *P*<0.05. IBM SPSS version 25 software (IBM Corp.) was used for statistical analyses.

## Results

### Patient characteristics

A total of 307 patients with thyroid disease underwent thyroid operation. Benign histopathology was reported in 187 cases (61%), and malignant histopathology in 120 cases (39%).

The mean age was 41.5±12.1 years (range: 19.0–71.0 years). The most typical age group was 30–39 years (*n*=104; 33.9%), followed by 40–49 years (*n*=89, 29.0%). Most cases were female (*n*=245, 79.8%) patients. Comorbidities, including hypothyroidism, hypertension, and diabetes, were presented in 53 (17.3%), 23 (7.5%), and 34 (11.1%), respectively. Previous thyroid surgery was reported in 10 (3.3%) patients. All the patients underwent blood laboratory investigation, US, and FNAC before surgery. The most commonly performed surgery was total thyroidectomy in 224 (73.0%) patients. Other procedures were right hemithyroidectomy, left hemithyroidectomy, and completion thyroidectomy in 41 (13.4%), 32(10.4%), and 10 (3.3%), respectively (Table 1).

**Table 1:** Patients’ clinicopathological characteristics (*N*=307).

Variables	*N* (%)
Age (years), Mean±SD	41.5±12.1 (range: 19.0–71.0)
Age groups	
Less than 20 years	8 (2.6%)
Between 20 and 30 years	35 (11.4%)
Between 30 and 39 years	104 (33.9%)
Between 40 and 49 years	89 (29.0%)
Between 50 and 59 years	45 (14.7%)
Between 60 and 69 years	17 (5.5%)
More than 70 years	9 (2.9%)
Sex	
Male	62 (20.2%)
Female	245 (79.8%)
Comorbidities	
History of hypothyroidism	53 (17.3%)
History of diabetes	34 (11.1%)
History of hypertension	23 (7.5%)
Family history of cancer	18 (5.9%)
History of asthma	13 (4.2%)
History of hyperthyroidism	12 (3.9%)
History of ischemic heart disease	3 (1.0%)
History of hormone therapy	2 (0.7%)
History of radiation therapy	1 (0.3%)
History of previous thyroid surgery	10 (3.3%)
ASA classification	
ASA 2	172 (56.0%)
ASA 1	107 (34.9%)
ASA 3	28 (9.1%)
Type of operation	
Total thyroidectomy	137 (44.6%)
Total thyroidectomy and lymph node neck dissection	87 (28.4%)
Right hemithyroidectomy	41 (13.4%)
Left hemithyroidectomy	32 (10.4%)
Completion thyroidectomy	10 (3.3%)
Final histopathology	
Benign thyroid histopathology	187 (61%)
Goiter	100 (32.6%)
Toxic multinodular goiter	39 (12.7%)
Thyroiditis (Graves, Hashimoto’s)	20 (6.5%)
Thyroid cyst	9 (2.9%)
Benign thyroid nodule	47 (15.3%)
Follicular nodule	47 (15.3%)
Malignant thyroid histopathology	120 (39%)
Follicular thyroid neoplasm	47 (15.3%)
Medullary thyroid neoplasm	1 (0.3%)
Papillary thyroid cancer	67 (21.8%)
Hurthle cell neoplasm	2 (0.7%)
Lymphoma	1 (0.3%)
Carcinoma with mixed features	1 (0.3%)
Metastatic carcinoma	1 (0.3%)

ASA, American Society of Anesthesiologists.

The final histopathology report was benign histopathology in 187 (61%) patients that included goiter, toxic multinodular goiter, thyroiditis (graves, Hashimoto’s), thyroid cyst, benign thyroid nodule, and follicular nodule in 100 (32.6%), 39 (12.7%), 20 (6.5%), 9 (2.9%), 47 (15.3%), and 47 (15.3%), respectively. Malignant histopathology was reported in 120 (39%) patients, including papillary thyroid cancer and follicular thyroid neoplasm in 67 (21.8%) and 47 (15.3%) patients, respectively. Other malignant type was medullary thyroid neoplasm in 1 (0.3%) patient, Hurthle cell neoplasm in 2 (0.7%) patients, lymphoma in 1 (0.3%) patient, carcinoma with mixed features in 1 (0.3%) patient, and metastatic carcinoma in1 (0.3%) patient.

### Fine-needle aspiration cytology

#### Distribution of cases according to Bethesda categories

Patients were classified into various Bethesda categories, with 7 (2.3%) belonging to category I, 105 (34.2%) belonging to category II, 14 (4.6%) belonging to category III, 111 (36.2%) belonging to category IV, 45 (14.7%) belonging to category V, and 25 (8.1%) belonging to category VI. We found that two (1.7%) of malignant cases were reported in Bethesda Category I, 12 (10.0%) in Bethesda Category II, 2 (1.7%) in Category III, and 36.7% in Category IV. In comparison, 10 (5.3%) benign cases were reported in Bethesda Category V. However, Bethesda Category VI correctly reported all malignant cases and was statistically significant (*P*<0.001) (Table 2).

**Table 2:** Distribution of cases according to fine-needle aspiration cytology Bethesda and risk of malignancy based on postoperative report.

	FNAC	Pathology report^a^		
Bethesda classification	Total^b^	Benign (*N*=187, 61%)	Malignant (*N*=120, 39%)	*P*
I. Nondiagnostic or unsatisfactory	7 (2.3)	5 (2.7)	2 (1.7)	<0.001
II. Benign	105 (34.2)	93 (49.7)	12 (10.0)	
III. Atypia of undetermined significance or follicular lesion of undetermined significance	14 (4.6)	12 (6.4)	2 (1.7)	
IV. Follicular neoplasm or suspicious for a follicular neoplasm	111 (36.2)	67 (35.8)	44 (36.7)	
V. Suspicious for malignancy	45 (14.7)	10 (5.3)	35 (29.2)	
VI. Malignant	25 (8.1)	0 (0.0)	25 (20.8)	

^a^ The total number and percentage of patients based on postoperative pathology reports.

^b^ The total number and percentage of patients based on fine-needle aspiration cytology Bethesda class.

#### Correlation of FNAC with histopathological findings

**Figure 1:**
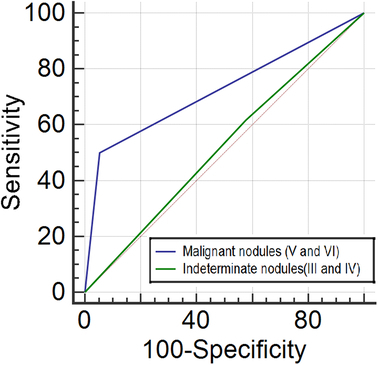
The area under the ROC curve indicates no discrimination for indeterminate nodules (III and IV) (AUC: 0.520; 95% CI: 0.462–0.577, *P*=0.495) and acceptable discrimination for malignant nodules (V and VI) (AUC: 0.723; 95% CI: 0.670–0.773, *P*<0.0001) based on the Bethesda classification.

Sensitivity, specificity, PPV, NPV, diagnostic accuracy, and AUC were determined individually for Bethesda categories III and IV (indeterminate nodules) and V and VI (malignant nodules). For indeterminate nodules, the sensitivity, specificity, PPV, NPV, and accuracy were 38, 58, 37, 59, and 50%, respectively. The ROC curve showed no significant discrimination, with an area under the curve of 0.520 (95% CI: 0.462–0.577, *P*=0.495), indicating no significant difference between the two groups (Fig. 1). For malignant nodules (V and VI), the sensitivity, specificity, PPV NPV, and accuracy were 50, 95, 86, 75, and 77%, respectively. The ROC curve indicates acceptable discrimination, with a statistically significant area under the curve (AUC: 0.723; 95% CI: 0.670–0.773, *P*<0.0001) (Fig. 1) (Table 3).

**Table 3:** Correlation of fine-needle aspiration cytology with histopathological findings.

Variables	Sensitivity	Specificity	PPV	NPV	Accuracy	AUC	*P*
Indeterminate nodules (III and IV) 125 (40.8%)	38% (95% CI: 48–30%)	58% (95% CI: 65–50%)	37%	59%	50%	0.520 (95% CI: 0.462–0.577)	0.495
Malignant nodules (V and VI) 70 (22.8%)	50% (95% CI: 59–41%)	95% (95% CI: 97–90%)	86%	75%	77%	0.723 (95% CI: 0.670–0.773)	**<0.0001**

Note: Boldface indicates a statistically significant result (*P*<0.05).

AUC, area under the curve; FNAC, fine-needle aspiration cytology; NPPV, negative predictive value; PPV, positive predictive value.

Ultrasonography findings: The US findings are summarized in Table 4.

**Table 4:** Sensitivity, specificity, positive predictive value, negative predictive value, and accuracy for fine-needle aspiration cytology according to FNAC Bethesda classification and ultrasonography features with surgical removal pathological result (*N*=307).

Variables	Subgroups	Total, *N* (%)^a^	Benign, *N* (%)^b^	Malignant, *N* (%)^c^		Sensitivity, % (95 CI)	Specificity, % (95 CI)	PPV, %	NPV, %	Accuracy, %	*P*
Ultrasonography findings											
Echogenicity	Hyperechoic	223 (72.6)	164 (87.7)		59 (49.2)	51% (95% CI: 60–42%)	88% (95% CI: 92–82%)	73%	74%	73%	**0.004**
	Hypoechoic	84 (27.4)	23 (12.3)		61 (50.8)						
Composition	Cystic or Spongiform	250 (81.4)	159 (85.0)	91 (75.8)		24% (95% CI: 33–17%)	85% (95% CI: 90–79%)	51%	64%	61%	0.405
	Solid	57 (18.6)	28 (15.0)	29 (24.2)							
Homogeneity	Homogenous	123 (40.1)	81 (43.3)	42 (35.0)		65% (95% CI: 73–56%)	43% (95% CI: 51– 36%)	42%	66%	52%	**0.004**
	Heterogenous	184 (59.9)	106 (56.7)	78 (65.0)							
Size	Less than 4 cm	247 (80.5)	145 (77.5)	102 (85.0)		15% (95 CI: 23–9%)	7 8% (95% CI: 83–71%)	30%	59%	53%	**0.064**
	More than 4 cm	60 (19.5)	42 (22.5)	18 (15.0)							
Width	Wider-than-tall	215 (70.0)	175 (93.6)	40 (33.3)		67% (95% CI: 75–57%)	94% (95% CI: 97–89%)	87%	81%	83%	**0.001**
	Taller-than-wider	92 (30.0)	12 (6.4)	80 (66.7)							
Margin Status	Regular border	291 (94.8)	183 (97.9)	108 (90.0)		10% (95% CI: 17–5%)	98% (95% CI: 99–95%)	75%	63%	64%	**0.019**
	Irregular margins	16 (5.2)	4 (2.1)	12 (10.0)							
Calcifications	Negative or Large comet tail artifacts	244 (79.5)	164 (87.7)	80 (66.7)		18% (95% CI: 26–12%)	95% (95% CI: 97–90%)	69%	64%	65%	**0.028**
	Peripheral/rim calcifications	63 (20.5)	23 (12.3)	40 (33.3)							
Thyroid Extension	No thyroidal extension	275 (89.6)	177 (94.7)	98 (81.7)		33% (95% CI: 43–25%)	88% (95% CI: 92–82%)	63%	67%	66%	0.675
	Extra thyroidal extension	32 (10.4)	10 (5.3)	22 (18.3)							
Total US features	Mean±SD	1.9±1.3	1.3±1.0	2.8±1.2		88.33 (95% CI: 81.2–93.5)	63.10 (95% CI: 55.8–70.0)	60.6%	89.4%		<0.001
Ultrasonography findings with FNAC findings											
Combination of US and FNAC^d^	Mean±SD	2.5±1.5	1.7±1.1	3.7±1.2		83.33% (95% CI: 75.4–89.5)	79.14% (95% CI: 72.6–84.7)	71.9%	88.1%		<0.001

The boldface indicates a statistically significant result (*P*<0.05).

a The total number and percentage of patients based on US features.

b The total number and percentage of benign cases based on postoperative pathology reports.

c The total number and percentage of malignant cases based on postoperative pathology reports.

d Note: Only Bethesda Classification Malignant (V and VI) is included.

FNAC, fine-needle aspiration cytology; NPPV, negative predictive value; PPV, positive predictive value; US, Ultrasonography.

Echogenicity: The echogenicity was hyperechoic in 164 (87.7%) cases. Malignant cases, on the other hand, demonstrated hyperechoic and hypoechoic echotexture in 59 (49.2%) and 61 (50.8%) of the cases, respectively. The sensitivity, specificity, PPV, NPV, and accuracy rates were 51% (95% CI: 60–42%), 88% (95% CI: 92–82%), 73, 74, and 73%, respectively.

Composition: The composition was cystic or spongiform in 159 (85.0%) benign cases. Malignant cases, on the other hand, had cystic and solid echotexture in 91 (75.8%) and 29 (24.2%) instances, respectively. The sensitivity, specificity, PPV, NPV, and accuracy were 24% (95% CI: 33–17%), 85% (95% CI: 90–79%), 51, 64, and 61%, respectively.

Homogeneity: The echogenicity was uniform in 81 (43.3%) benign cases. Malignant cases, on the other hand, exhibited homogeneous and heterogeneous echotexture in 42 (35.0%) and 78 (65.0%) instances, respectively. The sensitivity, specificity, PPV, NPV, and accuracy were 65% (95% CI: 73–56%), 43% (95% CI: 51–36%), 42, 66, and 52%, respectively.

Nodule size: In 145 (77.5%) benign cases, nodules were smaller than 4 cm, while 18 (15.0%) malignant cases had larger dimensions (>4 cm). The sensitivity, specificity, PPV, NPV, and accuracy were 15% (95% CI: 23–9%), 7.8% (95% CI: 83–71%), 30, 59, and 53%, respectively.

Width: In 175 (93.6%) benign instances, the diameter of the thyroid tumor exceeded its height. Malignant cases were categorized as wider-than-tall in 40 (33.3%) and taller-than-wider in 80 (66.7%). The sensitivity, specificity, PPV, NPV, and accuracy were 67% (95% CI: 75–57%), 94% (95% CI: 97–89%), 87, 81, and 83%, respectively.

Margin: In 183 (97.9%) benign cases, regular margins were found. One hundred eight (90.0%) had regular margins, and 12 (10.0%) had irregular margins. The sensitivity, specificity, PPV, NPV, and accuracy rates were 10% (95% CI: 17–5%), 98% (95% CI: 99–95%), 75, 63, and 64%, respectively.

Calcifications: Calcifications were absent in 164 benign cases (87.7%) and present in 80 malignant cases. The sensitivity, specificity, PPV, NPV, and accuracy were 18% (95% CI: 26–12%), 95% (95% CI: 97–90%), 69, 64, and 65%, respectively.

Thyroid extension: Thyroid extension was observed in 10 (5.3%) and 22 (18.3%) benign and malignant patients. The sensitivity, specificity, PPV, NPV, and accuracy were 33% (95% CI: 43–25%), 88% (95% CI: 92–82%), 63, 67, and 66%, respectively.

**Figure 2:**
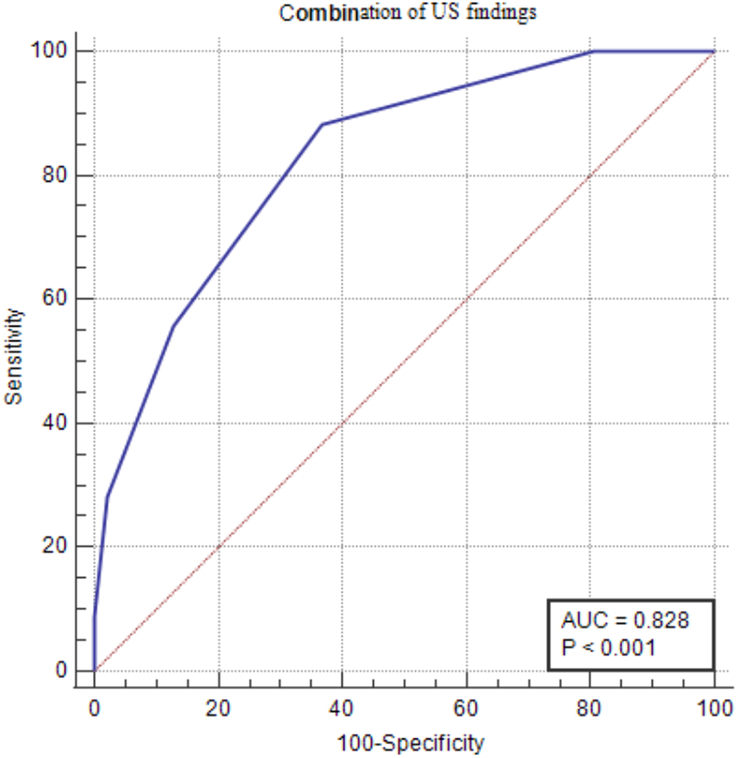
The area under the ROC curve for a combination of US parameters showed good agreement (AUC: 0.828; 95% CI: 0.781–0.868, *P*<0.001) with sensitivity, specificity, PPV, and NPV of 88.33, 63.10, 60.6, and 89.4%, respectively.

Correlation of US findings with histopathological findings: A combination of US findings provided a sensitivity, specificity, PPV, and NPV of 88.33% (95% CI: 81.2–93.5), 63.10% (95% CI: 55.8–70.0), 60.6, and 89.4%, respectively. The ROC curve exhibited good agreement and was statistically significant (AUC: 0.828; 95% CI: 0.781–0.868, *P*<0.001) (Fig. 2). Furthermore, univariate analysis revealed that hypoechoic appearance, heterogenicity, big mass size (more than 4 cm), taller-than-wider, infiltrative margins, and microcalcifications were risk factors for thyroid nodule malignancy based on final histopathology results.

**Figure 3:**
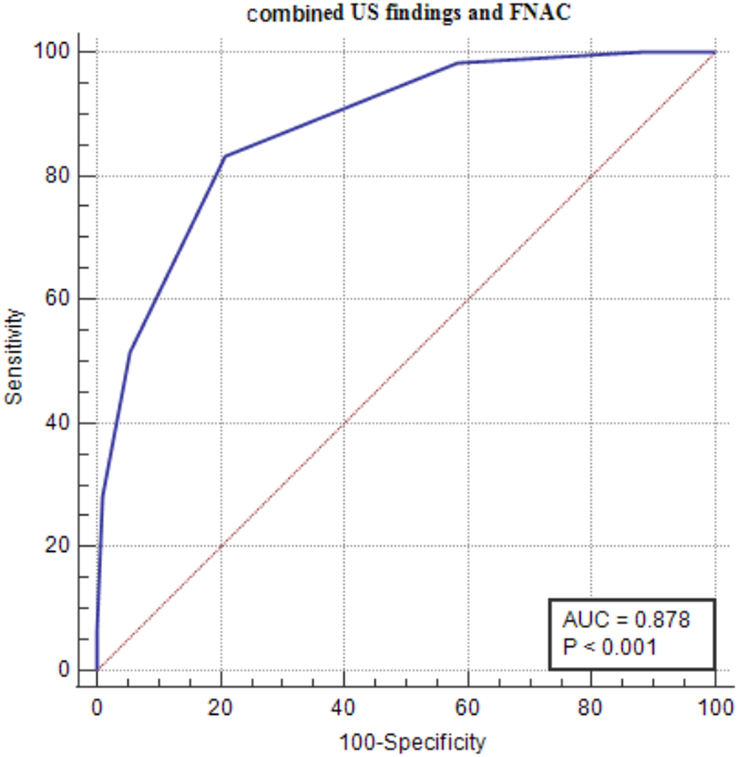
The area under the ROC curve of the combined use of US parameters and FNAC yielded a significantly higher AUC value of 0.878 (95% CI: 0.836–0.913; *P*<0.0001) with a higher sensitivity of 83.33 and specificity of 79.14%.

Correlation of combined US and FNAC results with histopathological findings: ROC curve analysis was performed, including FNAC and other quantitative US measurements. This analysis was done to see how they correlated with the findings from postoperative histology. The combination of these parameters resulted in a considerably higher AUC value of 0.878 (95% CI: 0.836–0.913; *P*<0.0001) in ROC curve analysis. This combination also produced a greater sensitivity of 83.33% (95% CI: 75.4–89.5) and specificity of 79.14% (95% CI: 72.6–84.7) than the separate parameters (Fig. 3) (Table 4).

## Discussion

Thyroid nodule diagnosis is challenging due to limited clinical evaluations and the need for invasive diagnostic methods like FNAC or surgery to rule out malignant processes^14,15^. Our study evaluated the diagnostic accuracy of US parameters and FNAC, individually and in combination with the postoperative histopathological examination, which is considered the gold standard in diagnostic testing. The latest thyroid guidelines urge the US for all patients with a suspected thyroid nodule. However, significant ambiguity exists over the diagnostic accuracy of US characteristics used to indicate the malignant potential of thyroid nodules^14^. A meta-analysis suggests that US features in isolation did not provide reliable information, and a combination of US characteristics is associated with higher likelihood ratios, indicating a higher risk of malignancy, which is more accurate for selecting nodules for FNAC^16^. The findings aligned with our findings, where a combination of US findings yielded a high sensitivity, specificity, PPV, and NPV of 88.33, 63.10, 60.6, and 89.4%, with a statistically significant area under the ROC curve. (AUC: 0.828 and *P*<0.001). Furthermore, our result aligns with the correlation recognized in the existing literature^16–20^.

In this study, we found that hypoechoic appearance, heterogenicity, large nodule (>4 cm), taller than wider shape, irregular nodule margins, and microcalcifications were risk factors for malignancy in thyroid nodules in univariate analysis, similar to Gu *et al*.^20^ study, which found malignant nodules more commonly present with large nodules (diameter >10 mm), solid structure, infiltrative margins, hypoechoic appearance, and microcalcifications. Doubi *et al*.^21^ discovered that hypoechogenic nodules, irregular margins, hypervascular or penetrating vessel vascularization, and a positive or complete fine halo sign were all risk factors for thyroid nodule cancer. In other studies, microcalcification, hypoechogenicity, cystic element, ‘taller-than-wide’, and dissimilar borders are highly associated with the malignant potential of the thyroid nodule, such as Boudina *et al*.^13^ Nabahati *et al*.^22^, and Ram *et al*.^23^. Li *et al*.^24^ found that solid portions ≥50%, eccentric solid portions, irregular nodule shape, microcalcification, and intraocular vascularity were significantly associated with thyroid cancer based on FNAC cytology findings. Kwak *et al*.^25^ discovered that the following US characteristics were significantly associated with malignancy: solid component, hypoechogenicity, pronounced hypoechogenicity, micro-lobulated or irregular edges, microcalcifications, and taller-than-wide shape based on FNAC cytology findings.

Our study found that malignant nodules were more likely to have calcification than benign ones, and microcalcification was associated with thyroid carcinoma. The sensitivity, specificity, PPV, NPV, and accuracy of microcalcification were 18, 95, 69, 64, and 65%, similar to Gu *et al*.^20^ findings, but they reported a higher sensitivity (65.5%). Wang *et al*.^26^ discovered that microcalcifications and uneven or micro-lobulated margins were independently related to malignancy. Microcalcifications and colloid crystals may be challenging to distinguish in small nodules under 10 mm, reducing US accuracy. We also found that malignant cases displayed solid echotexture in 29 (24.2%) cases with sensitivity, specificity, PPV, NPV, and accuracy rates were 24, 85, 51, 64, and 61%, respectively. These findings were aligned with the correlation recognized in the existing literature, such as Gu *et al*. and Li *et al*., but with low PPV (15–27%) in the Li *et al*. report^20,27^.

Our study found that US traits such as taller-than-wider forms and hypoechogenicity had the highest diagnostic accuracy in identifying thyroid nodule patterns (83 and 73%, respectively). Wang *et al*.^26^, utilizing distinct US (TI-RADS), found that marked hypoechogenicity was the most important independent predictor of malignancy, similar to the findings presented. Furthermore, Woo *et al*.^28^ found that hypoechogenicity was the sole statistically significant feature in the US. Remonti *et al*.^16^ discovered that microcalcifications, uneven edges, and a taller-than-wide form exhibited high specificities for malignant nodule detection.

Our study found that combining US parameters and FNAC results improved the test’s AUC value, sensitivity, and specificity compared to individual parameters. The findings were consistent with the correlations established in previous literature, including those by Ghabisha *et al*. and Li *et al*.^10,11^. Li *et al*. discovered that FNAC combined with the US significantly improved the sensitivity and accuracy of thyroid nodule diagnosis in thyroid lesions less than 1 cm. Still, it had no significant effect on accuracy, sensitivity, or specificity in thyroid nodule diagnosis in group thyroid lesions larger than 1 cm; the sensitivity was 100%, and the specificity was 63.64%^10^. In another report, the US score had higher diagnostic accuracy and sensitivity than the Bethesda score, but both systems had comparable specificities and PVV^13^. Boudina *et al*. found that the diagnostic accuracy of the US score had higher sensitivity (64.2 vs. 33.3%) and a better NPV (34.5 vs. 24.4%) than the Bethesda score. However, both scoring systems had comparable specificities (90.9 vs. 100%) and PPV (97.1 vs. 100%)^13^.

FNAC is a diagnostic tool capable of identifying benign or malignant thyroid nodules, with a sensitivity of 65–99% and a specificity of 72–100%^29^. Our study found that Bethesda Category VI correctly reported all malignant cases, while Bethesda Category I to IV reported 1.7, 10.0, 1.7, and 36.7% of malignant cases. Furthermore, 5.3% of benign cases were reported in Bethesda Category V. The malignancy rates were observed in Osseis *et al*.^17^ report for categories I to VI were 16.67, 20, 54.54, 34.21, 88.89, and 100%, respectively. We also found acceptable discrimination for malignant nodules (AUC: 0.723 and *P*<0.0001), with sensitivity, specificity, PPV NPV, and accuracy of 50, 95, 86, 75, and 77%, respectively. In another study, Boudina *et al*.^13^ found that FNAC demonstrated specificity and PPV of 100%, while its sensitivity was as low as 33%, and its diagnostic accuracy was 43.75%. In another report of FNAC, Hirachand *et al*.^30^ found a sensitivity of 52.6%, specificity of 86.6%, and accuracy of 79.1%. In contrast, Hisham *et al*. and Shah *et al*. found that FNAC has high accuracy, sensitivity, specificity, PPV, and NPV for thyroid cancer prediction^6,9^. This variation of FNAC reports may be attributed to false positives and negative reports due to potentially misinterpreting cystic degeneration and squamous cells or underestimating architectural and cellular features in follicular patterns^29^.

### Study limitations

The study has significant drawbacks. Firstly, the study collected retrospective data from patients’ medical records, which is prone to selection and misclassification bias. Secondly, operator expertise can alter the metrics employed, such as US and FNAC, and false-negative cases may impair the accuracy of the effect estimate. Thirdly, the study also encountered problems with parameter cutoffs, low repeatability, and heterogeneity in results. Fourthly, because of its retrospective nature, US characteristics were examined using previously recorded images taken by diverse radiologists utilizing a range of equipment; nonetheless, all US and FNAC were conducted by experienced radiologists. Consequently, future large-scale prospective profound studies are required to validate our findings.

## Conclusion

Combining US characteristics with FNAC results can afford the maximum analytical accuracy in distinguishing benign from malignant thyroid nodules. This strategy is practical due to its simplicity, minimal invasiveness, and cost-effectiveness, enabling robust management regimens and avoiding additional surgical procedures.

## Ethical approval

Ethical approval for this study (Ethical Committee N° 2024-5 E) was provided by the Research Ethics Committees of King Khalid Hospital, Najran, Saudi Arabia on 17 April 2024.

## Consent

Written informed consent was obtained from the patient for publication and any accompanying images. A copy of the written consent is available for review by the Editor-in-Chief of this journal on request.

## Source of funding

This research received no external funding.

## Author contribution

M.A.: conceptualization; M.A.-M. and M.A.M.: methodology; A.S., F.A., R.A., and A.A.: software; M.A., F.A., and A.A.: validation; F.A., M.A., M.A., and A.S.: formal analysis; M.A., A.S., F.A., and M.A.: investigation; A.A. and M.A.: resources; M.A. and M.A.: data curation; F.A., A.S., R.A., and A.M.: writing – original draft preparation; M.A.M. and A.A.: writing – review and editing; F.A.: visualization; M.A.: supervision; M.A.-M: project administration. All authors have read and agreed to the published version of the manuscript.

## Conflicts of interest disclosure

The authors declare no conflicts of interest.

## Research registration unique identifying number (UIN)

1. Name of the registry: UMIN Clinical Trials Registry.
2. Unique Identifying number or registration ID: TEST000001927.
3. Hyperlink to your specific registration (must be publicly accessible and will be checked): https://center6.umin.ac.jp/cgi-openbin/ctr_e/ctr_view.cgi?recptno=T000009482.

## Guarantor

Mana Alhajlan and Faisal Ahmed.

## Data availability statement

The data presented in this study are available on request from the corresponding authors. The data are not publicly available due to local policies.

## Provenance and peer review

Not commissioned, externally peer-reviewed.
